# Platelets in infection: intrinsic roles and functional outcomes

**DOI:** 10.3389/fimmu.2025.1616783

**Published:** 2025-07-07

**Authors:** Yuwei Hu, Shuchang Dai, Congchao Qiao, Yifan Ye, Junyi Ren, Kai Wang, Ling Li, Zhong Liu

**Affiliations:** ^1^ Clinical Transfusion Research Center, Institute of Blood Transfusion, Chinese Academy of Medical Sciences & Peking Union Medical College, Chengdu, China; ^2^ Key Laboratory of Transfusion Adverse Reactions, Chinese Academy of Medical Sciences, Chengdu, China; ^3^ Department of Transfusion Medicine, Dazhou Integrated Traditional Chinese Medicine and Western Medicine Hospital, Dazhou Second People’s Hospital, Dazhou, Sichuan, China; ^4^ State Key Laboratory of Experimental Hematology, National Clinical Research Center for Blood Diseases, Institute of Hematology and Blood Diseases Hospital, Chinese Academy of Medical Sciences and Peking Union Medical College, Tianjin, China; ^5^ School of Medicine, University of Electronic Science and Technology of China, Chengdu, Sichuan, China; ^6^ Department of Emergency, Sichuan Provincial People’s Hospital, University of Electronic Science and Technology of China, Chengdu, China; ^7^ Department of Blood Transfusion, The Third People’s Hospital of Chengdu (Affiliated Hospital of Southwest Jiaotong University), College of Medicine, Southwest Jiaotong University, Chengdu, Sichuan, China; ^8^ School of Public Health, Anhui Medical University, Hefei, China

**Keywords:** platelets, infection, immunoregulation, immunothrombosis, pathogen clearance, immune response, immunity, host defense

## Abstract

Platelets have long been acknowledged for their essential roles in hemostasis and thrombosis; however, recent insights highlight their broader involvement as key participants in host responses during infection. Beyond their classical functions, platelets exhibit diverse anti-infective capabilities, such as direct pathogen internalization, receptor-mediated pathogen recognition, the release of antimicrobial peptides, cytokines, and chemokines, and the generation of immunomodulatory extracellular vesicles. These intrinsic platelet attributes enable dynamic interactions with pathogens and immune cells, significantly contributing to pathogen capture, neutralization, and the orchestration of innate and adaptive immune responses. This review examines the multifaceted intrinsic roles of platelets and delineates the beneficial outcomes of their activation, providing an integrated perspective on platelet-driven immunity and defense mechanisms during infection.

## Graphical Abstract

Overview of platelet activation, its intrinsic roles and functional outcomes during infection. This schematic illustrates the transition of platelets from the resting to activated state and their multifaceted involvement in infection. Upon activation, platelets execute four major functions: (1) formation of immunothrombosis involving fibrin, neutrophil extracellular traps (NETs), and *S. aureus* to restrict pathogen spread; (2) direct interactions and internalization with various pathogens including bacteria, fungi, and viruses; (3) release of antimicrobial substances via degranulation and generation of platelet-derived microparticles (PMPs) and exosomes (PL-EXOs); and (4) modulation of immune responses through immunomodulatory factor release, chemokine secretion, and direct interaction with leukocytes. These activities are grouped into three functional roles—coagulatory, anti-infective, and immunoregulatory—ultimately contributing to pathogen sensing and capture, promotion of pathogen clearance, and modulation of immune responses.

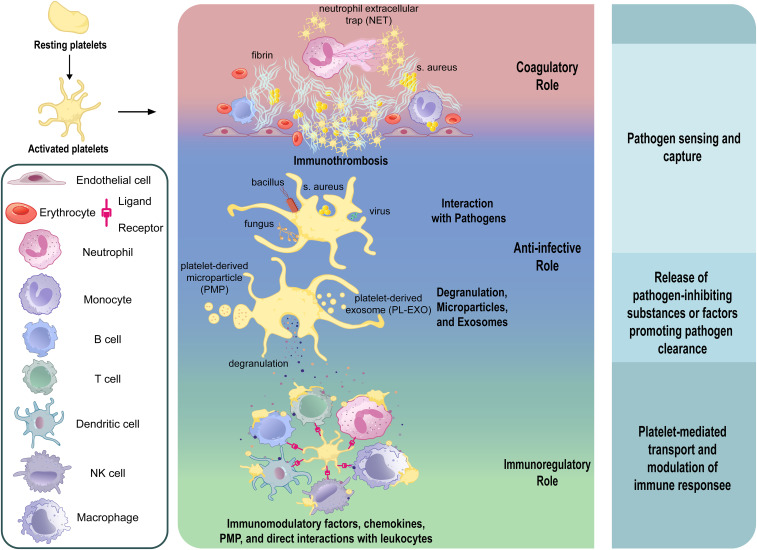

## Introduction

In recent years, accumulating evidence has highlighted that platelets actively participate in host immune responses—including pathogen recognition, inflammatory regulation, and tissue repair—alongside their classical roles in coagulation and thrombosis (1–7). They express an array of immune receptors, including pattern recognition receptors (PRRs) (8–11), adhesion molecules (12–15), and cytokine/chemokine receptors (16, 17), enabling platelets to directly sense and respond to pathogens and inflammatory stimuli (18–20). Upon activation by microbial components or inflammatory stimuli, platelets initiate immune defense by first capturing and internalizing pathogens through receptor-mediated recognition, targeting a broad range of microbes including bacteria, fungi, and viruses (4, 10, 21–27). They subsequently promote the formation of immunothrombosis, a coordinated intravascular response involving fibrin deposition, neutrophil extracellular traps (NETs), and platelet aggregation that serves to localize pathogens and prevent their systemic dissemination (28–30). Concurrently, activated platelets undergo degranulation, releasing antimicrobial peptides such as thrombocidins and defensins from α-granules, which exert direct microbicidal effects (31–34). In addition, platelets release platelet-derived microparticles (PMPs) and exosomes (PL-EXOs), which carry cytokines, chemokines, and immunomodulatory molecules, thereby amplifying the local immune response and facilitating crosstalk with other immune cells (35–37).

Clinically, platelet count and functionality have also emerged as significant prognostic markers in infectious diseases (38–41). Thrombocytopenia, commonly observed in severe infections and sepsis, is strongly associated with increased morbidity and mortality (39, 42–49). Additionally, disseminated intravascular coagulation (DIC), a severe complication characterized by systemic coagulation dysregulation commonly seen in advanced stages of sepsis, further underscores the critical interplay between platelets and immune-mediated pathological conditions (50, 51).

Physiologically, platelet counts range from 150 to 450 × 10^9^/L. However, in clinical contexts such as hematologic malignancies or allogeneic hematopoietic progenitor cell transplant, a threshold of approximately 10 × 10^9^/L is often sufficient for prophylactic platelet transfusion to prevent spontaneous bleeding (18, 52–55). This considerable functional reserve strongly suggests that platelets exert additional biological functions beyond hemostasis (18, 56–59).

Given that platelets exhibit intrinsic functional roles that extend beyond coagulation regulation to include coordinating immunological defense mechanisms during infection, understanding these diverse platelet roles and their functional outcomes in infectious diseases yields valuable insights that may inform the development of therapeutic interventions, such as platelet-mediated immunoregulatory, strategies to promote platelet regeneration, and transfusion guidance for managing thrombocytopenia in infectious conditions.

In this review, we summarize the multifaceted intrinsic roles of platelets and their functional outcomes and discuss the clinical implications of platelet depletion in infectious diseases.

## Methods

To systematically summarize existing scientific evidence regarding the roles of platelets during infection, we conducted a targeted literature review using PubMed, Web of Science (ISI), and the Chinese Biomedical Literature Database (CBM), covering both English and Chinese publications up to 1 April 2025. The search strategy employed Boolean logic to combine three conceptual domains: (1) platelets and platelet transfusion-related products, (2) infection and sepsis-associated conditions, and (3) immunological mechanisms and related cellular processes. Search terms included both MeSH terms and free-text keywords and are provided in the **Supplementary Material** (**Supplementary Table S1**).

After initial retrieval, duplicate records were removed using EndNote and manual verification. We then screened titles and abstracts to identify studies of relevance, prioritizing original research and high-quality review articles that explored the intrinsic roles and functional outcomes of platelets during infection. Full-texts of eligible articles were obtained. This review synthesizes the current evidence, aiming to provide an integrated perspective on the intrinsic roles and functional outcomes of platelet activation during infection.

## Roles

### Coagulatory role of platelets in infection

In infection, particularly sepsis, the coagulation cascade is systemically activated through pathogen-driven and platelet-mediated mechanisms, contributing to immune thrombosis and DIC (7, 50, 60–62). Platelets activated by microbial pathogen-associated molecular patterns (PAMPs), such as lipopolysaccharide (LPS), release procoagulant factors and facilitate thrombin generation on their surfaces, significantly enhancing clot formation. Concurrently, bacterial-induced tissue damage and inflammation upregulate tissue factor (63), initiating the extrinsic pathway of coagulation (64, 65). Additionally, pathogens trigger activation of coagulation factor XII (FXII) through PAMPs, facilitating the intrinsic coagulation pathway, while activated platelets enhance NETosis, further amplifying immunothrombosis (28, 29, 66). Notably, recent evidence from SARS-CoV-2 infection demonstrates that fibrin forms complexes with the viral spike protein, generating proinflammatory clots that drive oxidative stress, immune dysregulation, and neuronal injury in both the lungs and brain (30). Despite compensatory fibrinolytic responses aimed at resolving excessive fibrin deposition, pathological impairment of fibrinolytic activity sustains persistent thrombotic states, resulting in DIC, characterized by systemic microthrombosis and bleeding, ultimately progressing toward multi-organ failure (67).

### Anti-infective role of platelets in infection

Platelets exert multifaceted anti-infective capabilities through degranulation and interactions with pathogens, involving receptor-mediated binding, indirect interactions via plasma proteins, and rapid responses to microbial toxins and viral mediators.

#### Interactions with pathogens

##### Direct interactions

**Figure 1:**
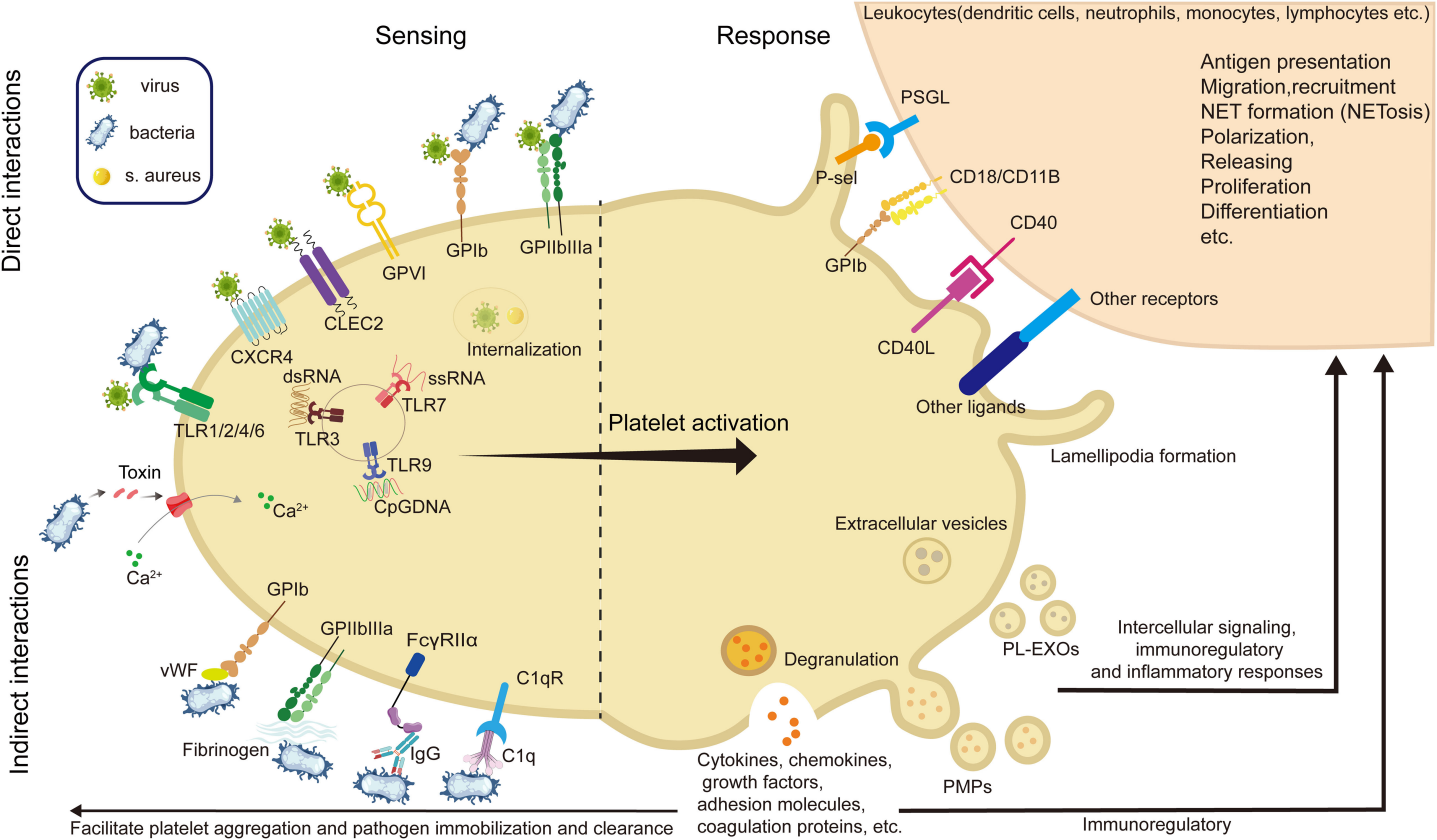
Diagram of platelet interactions with pathogens and downstream responses in infection. During the process of infection and inflammation, platelets sense pathogens such as bacteria and viruses through direct and indirect interactions, and respond by releasing granules and vesicles, and binding to immune cells, ultimately exerting anti-infective and immunoregulatory effects. (GPVI, glycoprotein VI; GPIb, glycoprotein Ib; GPIIb/IIIa, glycoprotein IIb/IIIa; CLEC-2, C-type lectin-like receptor 2; DC-SIGN, dendritic cell-specific intercellular adhesion molecule-3-grabbing non-integrin; TLR, toll-like receptor; FcγRII, Fc gamma receptor II; CD40L, CD40 ligand; MHC-I, major histocompatibility complex class I; NETs, neutrophil extracellular traps; PMP, platelet-derived microparticles; PL-EXOs, platelet-derived exosomes).

Platelets directly interact with pathogens through a broad repertoire of surface receptors and innate immune sensors, enabling rapid pathogen recognition, internalization, and immunoregulatory (**Figure 1**). For bacterial pathogens, classical integrins such as GPIIb-IIIa (68–70) and GPIbα (68, 71–73) mediate adhesion and internalization (**Figure 1**). IsdB protein of *Staphylococcus aureus (S. aureus)* (69, 74) and PadA of *Streptococcus gordonii* (*S. gordonii*) (70) directly bind GPIIb-IIIa independently of fibrinogen. GPIbα, part of a leucine-rich glycoprotein complex, binds bacterial proteins such as SrpA from *Streptococcus sanguinis* (72), GspB from *S. gordonii* (72), and SarP from S. aureus (75), facilitating pathogen capture and host defense. In addition, platelets recognize PAMPs through PRRs, including Toll-like Receptors(TLRs), C-type Lectin Receptors(CLRs), and NOD-like Receptors(NLRs) (76–79). TLR4 detects bacterial LPS, initiating platelet activation, cytokine release, and immune response modulation (80–86). Intracellular TLRs (TLR3 (87–89), TLR7 (10), TLR9 (90)) recognize viral nucleic acids, stimulating inflammatory responses (**Figure 1**). CLRs, notably CLEC2, mediate platelet activation critical in viral infections (91, 92), whereas NLRs, particularly NLRP3 and NOD2, enhance cytokine secretion and platelet functions upon pathogen detection (93, 94).

Furthermore, viral particles can also engage platelet integrins such as GPIIb and αVβ3, which facilitate adhesion and internalization, as observed with hantaviruses and adenoviruses (95–97). Other integrins, including α2β1, mediate binding to rotaviruses (98), while coxsackievirus B utilizes the Coxsackie and adenovirus receptor (CAR) for platelet interaction (99). Platelet GPVI, classically involved in collagen sensing, has been shown to recognize *hepatitis C virus* (HCV) (100). C-type lectin domain family 1-member B(CLEC-2) and Dendritic Cell-Specific Intercellular adhesion molecule-3-Grabbing Non-integrin(DC-SIGN) also serve as viral interaction points, particularly in the context of HIV and *dengue virus* (DENV) (101) (**Figure 1**). These multifaceted receptor-mediated interactions highlight the direct role of platelets in viral recognition and immune activation, complementing their antibacterial functions and reinforcing their importance in antiviral defense.

The internalization of bacteria by platelets was initially observed with *S. aureus*, demonstrated to occur independently of the open canalicular system (OCS) in vacuoles expressing activation markers CD62P and GPIIb-IIIa (21–24). Bacterial internalization mechanisms differ among pathogens; *S. aureus* requires platelet activation (e.g. by ADP or thrombin) for internalization, whereas *Porphyromonas gingivalis* (*P. gingivalis*) can independently induce its internalization by platelets via aggregation mechanisms alone (23). Similarly, Platelets internalize various viruses, including HIV (10), influenza virus (eg.H1N1) (4), DENV (102), and HCV (103, 104), and these interactions lead to distinct downstream consequences (4, 10, 25–27). Viral internalization typically triggers platelet activation and degranulation, contributing to viral clearance; however, in certain contexts, this process may instead favor viral persistence. For instance, Koupenova et al. observed HIV localized within platelet vacuoles that fuse with α-granules rich in inflammatory peptides, potentially facilitating viral degradation (10). In contrast, HCV internalization by platelets appears to shield the virus from immune clearance, limiting uncoating without promoting elimination (27). Platelets harboring DENV have shown negative-stranded viral RNA, suggesting the possibility of limited replication, though no productive infection or viral transmission to other cells has been documented (27). This duality—clearance versus concealment—likely depends on the virus type and host context, representing a promising but still underexplored area of platelet immunobiology with potential therapeutic implications.

##### Indirect interactions

In addition to direct receptor–ligand binding, platelets participate in pathogen recognition and clearance through a variety of indirect mechanisms mediated by plasma proteins and soluble immune components. Key among these are immunoglobulins, von Willebrand factor (vWF), fibrinogen, and components of the complement system (105–111).

Some GPIIb-IIIa binds to microbial surface components indirectly (21–24, 68–73, 112), recognizing adhesive matrix molecules (MSCRAMMs) (113), including ClfA (114), ClfB (115) and Fnbp (A and B) proteins (116) of *S. aureus*, and Fbl of *Streptococcus lugdunensis* (*S. lugdunensis*) (117). And GPIbα and GPIIb-IIIa, the principal platelet adhesion receptors, contribute to indirect pathogen engagement through bridging molecules such as vWF and fibrinogen, respectively (106, 118). *S. aureus* protein A binds to vWF, subsequently interacting with platelet GPIbα, thus promoting bacterial adhesion and aggregation (109). Glycoprotein-fibrinogen interactions further contribute to pathogen capture, involving various bacterial surface proteins such as ClfA (116), ClfB (114), FnbpA/B (117) from *S. aureus*, and Fbl from *S. lugdunensis* (115), each interacting with distinct fibrinogen domains.

Complement receptor interactions, including C1q receptor gC1q-R expressed on platelets, bind complement-coated pathogens, enhancing pathogen clearance while also marking platelets as targets for complement-mediated lysis under pathological conditions, emphasizing the dual immune and hemostatic roles of platelets (119–124). For instance, *Epstein-Barr virus* (EBV) binds platelet complement receptor CR2 (110), while HIV engages multiple receptors on platelets, including CXCR4, CCR1, CCR3, and CCR4, contributing to platelet activation and modulation of the antiviral response (111).

Similarly, platelets express FcγRIIa, a low-affinity receptor for IgG, which enables them to recognize and internalize IgG-opsonized pathogens, including SARS-CoV-2 (125). This internalization process facilitates fusion with α-granules that contain antimicrobial peptides, thereby promoting intracellular degradation of the pathogens (105–108, 126). Moreover, FcγRIIa mediates platelet responses to virus–antibody immune complexes (124, 127). In DENV infection, such immune complexes trigger platelet activation and aggregation via FcγRIIa, leading to efficient clearance of virus-coated platelets in the spleen, a key organ for platelet turnover and immune surveillance (128). Similarly, HCV infection, immune complexes are cleared through platelet-mediated mechanisms in the liver, underscoring the importance of tissue-specific immune environments in antiviral defense (100).

##### Microbial toxins and viral soluble mediators

Platelets play a critical role in host defense by responding to a variety of pathogen-derived toxins and soluble factors.

Several bacterial toxins directly interact with platelet membranes or receptors, leading to platelet activation and degranulation. For instance, α-toxin from *S. aureus* (129) and protease-activated receptor (PAR) activators from *P. gingivalis* (130) directly target platelet receptors, initiating cellular activation similar to thrombin. Other toxins, including pneumolysin from *Streptococcus pneumoniae* (*S. pneumoniae*) and streptolysin from *Streptococcus pyogenes* (*S. pyogenes*) interact with platelet membranes, potentially inducing platelet activation and degranulation, thus mediating innate defense mechanisms (106, 127, 131–133).

In viral infections, platelets can be activated indirectly via host-virus interaction intermediates. HIV-derived Trans-Activator of Transcription (TAT) protein binds to platelet CCR3 and integrin β3, promoting activation (101, 134). Additionally, the non-structural protein 1 (NS1) of dengue virus directly binds to platelet TLR4, inducing platelet activation and contributing to dengue-associated thrombocytopenia and hemorrhage (135).

In viral infections, platelets can be activated indirectly via host-virus interaction intermediates. HIV-derived Trans-Activator of Transcription (TAT) protein binds to platelet CCR3 and integrin β3, promoting activation (101, 134). Additionally, the non-structural protein 1 (NS1) of dengue virus directly binds to platelet TLR4, inducing platelet activation and contributing to dengue-associated thrombocytopenia and hemorrhage (135).

Collectively, these findings underscore the sensitivity of platelets to microbial and viral products and their participation in both proinflammatory and antiviral host responses. More details are summarized in **Table 1**.

**Table 1:** Examples of the interaction between platelets and pathogens.

Pathogen	Pathogen molecules	Bridging protein	Platelet receptor	Platelet response	Reference
Direct interactions					
	DAMPs	**-**	PRRs		
G^-^ bacterial	LPS	–	TLR4	Thrombocytopenia; Promote & inhibit platelet activation; Promote microvascular thrombosis in endotoxemia; induces the formation of NETs in liver and lung.	(25)
P. gingivalis	Lipopeptides	–	TLR1/2	Platelet PI3K/Akt activation, formation of platelet-neutrophil aggregates; lead to proinflammatory and proangiogenic responses	(136)
HCMV	Envelope glycoproteins	–	TLR2	Release of proinflammatory CD40L and interleukin-1β and proangiogenic vascular endothelial-derived growth factor.	(137)
Virus(influenza,HIV, HCV,EMCV, DENV. etc)	ssRNA/dsRNA	–	TLR3/7/9(intracellular)	Release of complement C3, mild thrombocytopenia and increased platelet-neutrophil aggregate formation, without any prothrombotic effect.	(25)
HIV-1,DENV, Salmonella Typhimurium (STm)		C1q	CLEC2/NLRP3/DC-SIGN	Activates platelets via CLEC2 to release extracellular vesicles (EVs), including exosomes (EXOs) and microvesicles (MVs).	(91–94)
coxsackievirus B			CAR	P-selectin and phosphatidylserine (PS) exposure	(138)
Streptococcus sanguinis/S. gordonii/S. aureus	SrpA/GspB/SarP	–	GPIb	platelets adhesion; the organism colonize; the expression of SraP appears to be a virulence determinant in endovascular infection.	(72, 75, 139)
S. aureus/S. gordonii /Staphylococcus epidermidis/hantaviruses/adenoviruses	IsdB/PadA/SdrG	–	GPIIbIIIa	platelet adhesion, dense granule secretion and platelet spreading; SdrG alone is sufficient to support platelet adhesion and aggregation through both direct and indirect mechanisms.	(70, 74, 140)
HIV			CXCR4/CCR1/CCR3/CCR4	platelet activation and modulation of the antiviral response.	(141)
HCV			GPVI	playing a role in viral transport and persistence.	(142)
Indirect interactions					
S. aureus	protein A	vWF	GPIb	Platelet adhesion and aggregation	(109)
S. aureus/S. lugdunensis	ClfA/ClfB/Fnbp A/B/Fbl	Fibrinogen	GPIIbIIIa	Stimulate rapid platelet aggregation; Infective endocarditis.	(117, 143)
SARS-Cov-2/HCV	spike protein	IgG	FcγRIIA	Enhanced platelet-mediated thrombosis.	(108, 125)
viral and bacterial pathogen	S. aureus protein A	C1q	gC1q-R/CR2	platelet and monocyte/neutrophil activation; marking platelets as targets for complement-mediated lysis under pathological conditions; enhancing pathogen clearance.	(127, 132, 133, 144–146)
Microbial toxins and viral soluble mediators					
S. aureus	α-toxin	–	–	α-toxin form transmembrane pores and trigger calcium mobilization to mediate platelet activation.	(129)
		–	ADAM10	Platelet intoxication prevents endothelial barrier repair and facilitates formation of injurious platelet-neutrophil aggregates	(79)
S. pneumoniae	pneumolysin	–	–	lyse platelets and to activate serum to become chemotactic.	(127, 132)
S. pyogenes	streptolysin	–	–	Activates human platelets via Streptolysin S-Mediated Calcium Ion influx	(131, 133)
P. gingivalis	Hgp44	–	PARs	The Hgp44 adhesin on the surface of bacterial cells is processed by Rgp and Kgp proteases and is necessary for platelet aggregation induced by Pseudomonas gingivalis in PRP.	(130)
Dengue virus	NS1 (nonstructural protein 1)	–	TLR4	Platelet activation, activating inflammasome to trigger IL-1β processing and release ATP.	(147)

### Degranulation

Upon activation, platelets release a spectrum of granule contents essential for their anti-infective functions. These granules include α-granules, dense granules, lysosomes, and specialized T-granules, each contributing distinct functions.

The α-granules, the most abundant platelet granules, contain various proteins, cytokines, chemokines, and growth factors, including adhesion molecules (GPVI, GPIIb/IIIa, GPIb-IX-V complex), coagulation proteins (e.g., vWF, fibrinogen, fibronectin), and cytokines/chemokines (e.g., platelet-derived growth factor [PDGF], transforming growth factor-β [TGF-β], CCL3, CCL5 (RANTES), and CXCL4/PF4 (31). These mediators facilitate not only platelet aggregation and pathogen immobilization but also immunoregulatory (see Section 3) (32, 33, 148). PF4 (CXCL4) binds to polyanionic structures on *E. coli*, undergoes conformational changes, and exposes neoepitopes that promote opsonization by anti-PF4/polyanion antibodies (128). PF4 also suppress HIV infection in some contexts (149, 150), while paradoxically enhancing HIV replication in others (151). CXCL7 is proteolytically cleaved into active fragments such as NAP-2 and connective tissue-activating peptide III (CTAP-III). Further C-terminal processing yields thrombocidins, a subclass of platelet-derived microbicidal peptides effective against *S. aureus*, *B. subtilis, E. coli*, and *L. lactis* (152). In addition, platelets also release Defensins, which exert direct antimicrobial effects upon release (153–155). For example, β-defensin 1 is stored in cytoplasmic compartments and released in response to *S. aureus* α-toxin (153), while α-defensin impairs the growth of *E. coli* (155). Platelets release antiviral peptides, such as PD1–PD4 and RW1–RW5, upon thrombin stimulation (156). Platelet-derived ROS and RNS (e.g., H_2_O_2_, NO_3_^-^, NO) also impair viral replication, including that of human cytomegalovirus (HCMV) (157).

Dense granules, containing smaller molecules such as ADP, serotonin, polyphosphates, histamine, and Ca²^+^, play a significant role in platelet activation and aggregation, thereby promoting rapid responses at infection sites (158). Platelet lysosomes, enriched with hydrolytic enzymes, are implicated in extracellular matrix degradation, receptor cleavage, and potentially autophagic clearance of pathogens (158). Additionally, platelets possess specialized T-granules containing TLR9, enabling recognition of bacterial DNA CpG sequences, thus actively participating in innate immune responses against pathogens (159).

## The immunoregulatory role of platelets in infection

### Platelet-derived immunoregulatory mediators

#### Soluble mediators and chemokines

Upon activation, platelets release a diverse array of immunomodulatory factors, including. vasoactive agents like serotonin (142) and platelet-activating lipids like TXA_2_ (160) and PAF (161), which modulate endothelial and immune cell activity. Growth factors such as PDGF (162) and TGF-β (141, 163–166) influence monocyte differentiation and lymphocyte regulation, while platelet-derived chemokines, including CXCL7, PF4, and CCL5, orchestrate neutrophil and monocyte recruitment, reinforcing host defense (167–170). Notably, PF4 also promotes the formation of neutrophil extracellular traps (NETs), thereby enhancing bacterial clearance (171), and supports antiviral immunity by recruiting leukocytes to the lungs during influenza infection (172) and modulating interferon responses during flavivirus infections such as dengue and Japanese encephalitis (173). Recognition of viral single-stranded RNA (e.g., from HIV or influenza) via TLR7 induces release of α-granule contents, CD40L, and P-selectin, also enhancing platelet-neutrophil aggregation and NET formation (174, 175). In parallel, activation of the NLRP3 inflammasome promotes cytokine secretion, while platelet-derived complement C3 amplifies neutrophil responses through further NET induction (4, 93, 119). Platelets also secrete antimicrobial peptides like thrombocidins and cytokines including IL-1β (146, 176), high mobility group box 1(HMGB1) (177, 178), and soluble CD40L ligand (sCD40L) (179) amplify immune signaling. As reservoirs of these immunomodulatory mediators, platelets not only regulate inflammation but also store and release a wide range of chemokines and cytokines that influence immune cell recruitment, wound healing, immune tolerance, and tumor metastasis (5, 111, 180, 181). Expressing multiple chemokine and cytokine receptors, platelets actively sense and respond to inflammatory signals, positioning them as key intermediaries linking innate and adaptive immunity.

#### Extracellular vesicles

Activated platelets also release extracellular vesicles classified into two main types: PMPs. and PL-EXOs (34), which are actively involved in immunoregulatory.

PMPs first reported in 1946 (182), represent the most abundant circulating microvesicle population, identifiable through enrichment via centrifugation and characterized by retained procoagulant activities (35, 36, 183). PMPs are heterogeneous vesicles released from the platelet plasma membrane, with diameters ranging from 100 nm to 1 µm (184). Under conditions of platelet activation or apoptosis, vesicles form through budding at specific sites of the cell membrane and eventually detach; some pseudopodia fragment, releasing debris into the bloodstream, thereby generating PMPs. This formation process involves calcium ion influx, phosphatidylserine exposure, and the Bax/caspase signaling pathway (185, 186). PMPs contain bioactive lipids (e.g., phosphatidylserine, tissue factor, arachidonic acid), surface proteins (e.g., CD41, CD31, P-selectin), and various microRNAs, supporting roles in coagulation, vascular repair, and immunoregulatory (187). Functionally, PMPs exert potent immunomodulatory effects. They activate neutrophils and endothelial cells via CD62P, enhancing neutrophil-endothelium adhesion (35), and facilitate monocyte recruitment through transfer of GPIbα (36). In viral infections, platelet activation by dengue virus (DENV) or SARS-CoV-2 triggers increased PMP release through CLEC-2 (91) or CD47 (188), respectively. These PMPs subsequently activate neutrophils and macrophages via TLR2 and TLR4 signaling, promoting NETosis and cytokine release, thereby amplifying inflammation (37). While PMP elevation is observed in many pathological states, the precise immunoregulatory mechanisms remain incompletely understood and merit further investigation.

PL-EXOs typically have diameters smaller than 100 nm, originating from early endosomes and multivesicular bodies (MVBs). They are released through fusion with the platelet cell surface and the OCS (184), a process dependent on ESCRT (138). PL-EXOs are enriched in tetraspanins (CD63, CD9, CD81) and endosomal sorting complex-related proteins (TSG101) (189). Additionally, exosomal cargo includes both mRNA and miRNA, exhibiting diverse types and abundant content (138). These vesicles mediate critical intercellular signaling, modulate immune and inflammatory responses, and facilitate tissue repair through miRNA delivery and receptor transfer (190, 191).

Moreover, PMPs and PL-EXOs can interact with distant tissues, including bone marrow, lymph nodes, and synovial fluids, highlighting their role in systemic communication during infections and other inflammatory conditions (138, 192).

### Platelet-mediated immune cell interactions

Platelets profoundly modulate immune responses through dynamic interactions with. innate and adaptive immune cells, facilitated primarily by direct cell-cell contacts and the release of immunomodulatory mediators. Platelets significantly modulate immune responses through interactions with various leukocytes, including dendritic cells (144, 145, 193), neutrophils (35, 71, 194, 195), monocytes (58), lymphocytes (196), even mast cell (197).

#### Innate immunity

**Figure 2:**
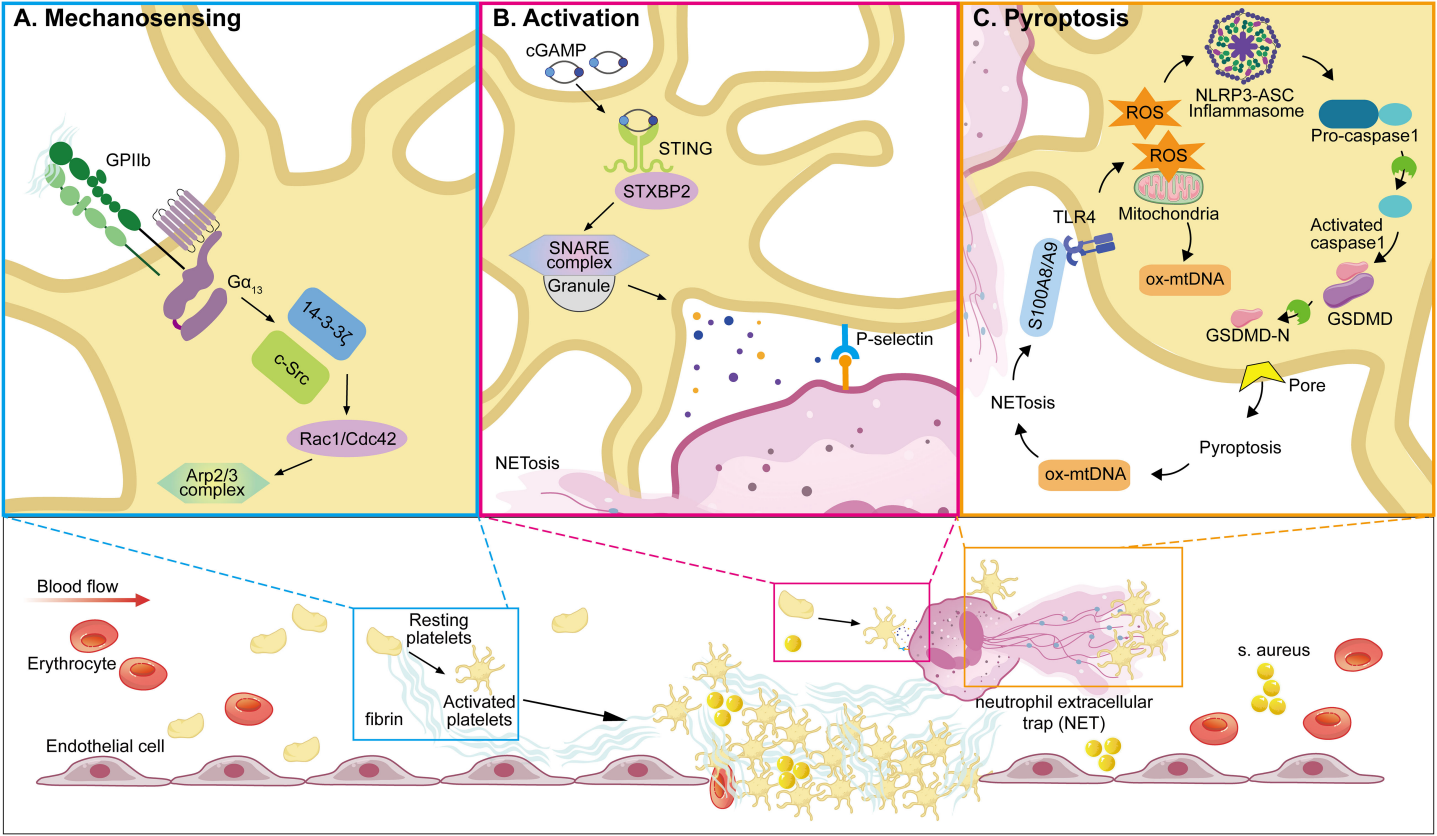
Representative molecular pathways involved in platelet resistance to infection. This illustration summarizes three recent reported, sequentially linked molecular pathways by which platelets participate in infection: **(A)** Mechanosensing-mediated migration (204). Upon vascular injury or inflammation, platelet integrin GPIIb senses fibrin(ogen)-rich matrix exposure and initiates mechanosensing through Gα13–c-Src–14-3-3ζ signaling. This promotes platelet polarization and lamellipodia formation, enabling directional migration toward sites of endothelial damage—a critical early step in immune hemostasis. **(B)** STING-dependent activation and NETosis (203). In sepsis, platelets are activated via STING (stimulator of interferon genes), which is triggered by cytosolic cGAMP or mitochondrial DNA. Activated STING interacts with the SNARE machinery through STXBP2 to promote granule secretion (e.g., P-selectin), facilitating platelet–neutrophil interactions and subsequent formation of NETs. **(C)** GSDMD-mediated pyroptosis (66). As inflammation escalates, platelet pyroptosis is induced through a S100A8/A9–TLR4–NLRP3 axis. Mitochondrial ROS trigger inflammasome assembly, leading to caspase-1 activation and cleavage of GSDMD. The resulting GSDMD-N fragments form membrane pores, causing pyroptotic cell death and the release of oxidized mitochondrial DNA (ox-mtDNA), which further enhances NET formation. NETs in turn release S100A8/A9, establishing a feedforward loop that amplifies platelet pyroptosis and inflammatory signaling. (GPIIb, glycoprotein IIb (integrin αIIb); Src, proto-oncogene tyrosine-protein kinase Src; 14-3-3ζ, 14-3–3 zeta protein; STING, stimulator of interferon genes; STXBP2, syntaxin-binding protein 2; SNARE, soluble N-ethylmaleimide-sensitive factor attachment protein receptor; cGAMP, cyclic GMP-AMP; NETs, neutrophil extracellular traps; TLR4, toll-like receptor 4; NLRP3, NOD-like receptor family pyrin domain-containing 3; GSDMD, gasdermin D; ox-mtDNA, oxidized mitochondrial DNA; ROS, reactive oxygen species; IL-1β, interleukin-1 beta; ASC, apoptosis-associated speck-like protein containing a CARD; PMNs, polymorphonuclear neutrophils).

Among innate immune cells, platelets play a pivotal role in regulating neutrophil. function. by forming platelet–neutrophil aggregates via P-selectin/P-selectin glycoprotein ligand 1(PSGL-1) and glycoproteins such as GPIbα and GPIIb, which promote neutrophil adhesion and transmigration to sites of inflammation (138, 185, 193, 194). They also induce neutrophil extracellular trap (NET) formation, a critical mechanism for trapping and neutralizing pathogens. In addition, platelet-derived chemokines like PF4 and CCL5 (RANTES) enhance neutrophil recruitment and activity, particularly under conditions such as acute lung injury (35, 71, 194, 195, 198–202). Recent studies have unveiled key mechanisms linking platelet activation to NETosis and immunothrombosis during sepsis. For instance, STING signaling in platelets has been shown to amplify granule secretion and intravascular thrombosis (203), while gasdermin D(GSDMD)-mediated platelet pyroptosis (66), driven by S100A8/A9–TLR4 signaling, promotes the release of oxidized mitochondrial DNA that enhances NET formation. These mechanisms establish a pathogenic feedback loop between platelets and neutrophils, contributing to excessive inflammation and tissue injury (**Figure 2**).

For monocytes, activated platelets form platelet–monocyte aggregates (PMAs) primarily via P-selectin–PSGL-1 binding, with co-stimulatory interactions involving CD40L–Mac-1 and GPVI–CD147,driving monocyte polarization toward an inflammatory M1 phenotype, amplifying cytokine secretion and contributing to immune activation in conditions like sepsis (58, 205–207). Conversely, in a murine model of systemic inflammation, the interaction of CLEC-2 on platelets with podoplanin on tissue-resident macrophages establishes an anti-inflammatory axis that mitigates immune cell infiltration and preserves vascular integrity during sepsis (208).

Platelets also engage with dendritic cells (DCs) through axes such as CD40–CD40L, P-selectin–PSGL-1, and JAM-C–Mac-1, promoting DC maturation, antigen internalization, and subsequent presentation to T lymphocytes—thereby bridging innate and adaptive immunity (144, 145, 168, 193, 209–213). Additionally, platelet-derived mediators such as CXCL4 and soluble CD40L (sCD40L) reinforce DC functions by upregulating costimulatory molecules and pro-inflammatory cytokines (210, 211).

Although less extensively studied, mast cells are another innate immune population influenced by platelet interaction (197). These interactions may modulate early inflammatory signaling and histamine release, further integrating vascular responses into immune defense.

#### Adaptive immunity

In adaptive immunity, platelets bridge innate signals and adaptive responses by directly, store and express substantial amounts of functional major histocompatibility complex class I (MHC-I) molecules, facilitating antigen processing and presentation to CD8+ T cells upon activation, thus enhancing adaptive immune responses (214). In T cells, platelet-derived CXCL4 (PF4) and serotonin are key immunoregulatory factors (5, 8, 215–217), while surface molecules such as CD62P, GPIIb/IIIa, and CD40L facilitate direct interaction with activated T lymphocytes (218–220). These interactions influence T-cell activation, proliferation, and trafficking to secondary lymphoid organs, with context-dependent outcomes—platelets may enhance or suppress T-cell effector functions depending on the immune context (216, 221–225). Moreover, platelets interact with B-cells via the CD40-CD40L axis, significantly affecting B-cell proliferation, differentiation, class switching, and memory formation, thereby modulating antibody-mediated immunity (199, 217, 226, 227). Conversely, platelets also contribute to inflammation resolution. In a murine model of bacterial pneumonia, they promote Treg expansion and macrophage polarization via TGFβ and CD62P-sCD40L interactions (179). Platelets further enhance T helper (Th)1, Th17 differentiation of CD4+ T cells via cell–cell contacts and release of PF4, CCL5 (RANTES) and TGFβ (223).

Furthermore, in shaping humoral immunity, platelet interactions with natural killer (NK) cells—mediated by immune checkpoint molecules like GITRL and RANKL—can suppress NK cell cytotoxicity and IFN-γ production, modulating immune responses under both infectious and neoplastic conditions (211, 212).

Interestingly, lymphocytes can also influence platelet biology: through the use of platelet-derived prostaglandin H_2_ (PGH_2_), lymphocytes synthesize PGI_2_, a potent inhibitor of platelet activation. This bidirectional regulation highlights the complex interplay between platelets and lymphocytes in shaping adaptive immunity (228, 229).

#### Transfusion-related immunomodulation

Instead of immunoregulatory functions of endogenous platelets, transfused allogeneic platelets can also exert potent immunomodulatory effects, which may be either beneficial or detrimental depending on the clinical context. TRIM refers to immune alterations associated with blood transfusions, encompassing immunosuppressive and pro-inflammatory effects potentially contributing to adverse clinical outcomes such as cancer recurrence, postoperative infections, multiorgan failure, and increased mortality (230, 231).

A large single-center cohort study demonstrated that allogeneic platelet transfusion during cardiac surgery was significantly associated with an increased risk of bloodstream infections, but showed no significant correlation with hospital-acquired pneumonia or surgical site infections. This suggests that platelet transfusion may selectively increase the risk of specific infections through immunomodulatory mechanisms (232). Kah et al. reported a case of an acute lymphoblastic leukemia patient who developed disseminated Fusarium infection and endogenous fungal endophthalmitis following leukocyte-depleted platelet transfusion. This supports the hypothesis that platelet-derived immunomodulatory factors may compromise host immune defenses through TRIM mechanisms, thereby increasing susceptibility to opportunistic infections (233). Regarding immunosuppressive effects, Sadallah et al. revealed that extracellular vesicles from stored platelets can redirect monocyte differentiation towards immature dendritic cells (iDC), which subsequently mature into DC, while simultaneously downregulating inflammatory responses in human macrophages (234). Using an *in vitro* whole-blood transfusion model, Perros et al. demonstrated that platelets modulate immune responses by suppressing DC-associated pro-inflammatory cytokines through soluble mediators while enhancing the anti-inflammatory cytokine IL-10 (235).

Multiple factors underlie TRIM, including the transfusion of allogeneic monocytes, soluble leukocyte-derived mediators, and circulating soluble HLA peptides within allogeneic plasma (230). Platelets have emerged as significant contributors to TRIM, with transfusion-associated platelet-derived molecules such as sCD40L, soluble OX40 ligand (sOX40L), soluble MHC class I (sMHC-I), and soluble FAS ligand (sFASL) increasing during storage and potentiating proinflammatory and immunomodulatory effects following transfusion (5, 6, 230, 236–242).

### Functional outcomes

#### Pathogen sensing and capture

Upon vascular injury in infection, platelet integrin GPIIb senses fibrin(ogen)-rich matrix exposure and initiates mechanosensing through Gα13–c-Src–14-3-3ζ signaling (204)(**Figure 2**). This promotes platelet polarization and lamellipodia formation, enabling directional migration toward sites of endothelial damage and forming immunothrombosis (204),which represents a critical component of the innate intravascular immune response, exerting multiple protective functions including pathogen containment, elimination, and immune coordination (243, 244). It achieves pathogen capture and confinement primarily through fibrin network formation within thrombi, preventing pathogen dissemination and tissue invasion (245). Additionally, the localized thrombotic environment supports innate immune cell recruitment and releases antimicrobial peptides at sites of pathogen entrapment, thereby enhancing pathogen clearance and immune defense mechanisms (3).

Platelets, which possess cellular structures facilitating virus attachment, internalization, and replication, further contribute to antiviral defense by sensing viral components through PRRs (3). Upon virus binding and internalization, platelets become activated, triggering granule secretion and promoting platelet-neutrophil interactions, which collectively enhance antiviral responses. For instance, in mouse models, platelets detect encephalomyocarditis virus via TLR7, resulting in significant platelet-neutrophil aggregate formation and rapid platelet consumption, leading to protective immunity (10). Similarly, during influenza infections, platelet-mediated virus internalization through TLR7 initiates the release of complement component C3, inducing neutrophil DNA release and aggregation, thus underscoring platelet-neutrophil crosstalk as a critical mechanism in orchestrating host immune and complement responses (4, 10).

### Platelet-mediated transport and induction of immune responses

Platelets significantly contribute to adaptive immune responses by facilitating pathogen transport and antigen presentation. Some platelet-bound bacteria persist in circulation long enough to be transported to the spleen, where they are recognized by CD8α+ dendritic cells, subsequently eliciting cytotoxic T-cell responses (246, 247). Similarly, in DENV infection, immune complexes trigger platelet activation and aggregation via FcγRIIa, leading to the efficient clearance of virus-coated platelets in the spleen, a key organ for platelet turnover and immune surveillance (128). In the case of HCV infection, immune complexes are cleared through platelet-mediated mechanisms in the liver (100).

In addition to transport, platelets influence pathogen fate via surface receptors. Platelet-expressed GPIb, for example, modulates the handling of pathogens opsonized by complement factor C3b. While C3b-coated bacteria are typically cleared by macrophages in the spleen, engagement by platelet GPIb redirects these complexes toward splenic dendritic cells, thereby enhancing adaptive immune responses (248, 249).

Moreover, megakaryocytes—precursors of platelets—express MHC class I molecules and actively process and cross-present antigens on their surface, initiating CD8+ T-cell activation and proliferation; during thrombopoiesis, these antigen-loaded MHC class I complexes are transferred to proplatelets (250). Given that platelets and megakaryocytes harbor all components necessary for antigen processing and presentation, platelets can directly interact with T-cells and also facilitate B-cell maturation and antibody class switching. Collectively, these mechanisms underscore the intricate interactions among platelets, antigen-presenting cells, and lymphocytes, highlighting the crucial role of platelets in orchestrating pathogen-specific adaptive immunity.

### Release of pathogen-inhibiting substances or factors promoting pathogen clearance

Upon activation, platelets release multiple substances that directly inhibit pathogen growth or facilitate pathogen clearance. Platelet-derived β-defensins, a group of cationic antimicrobial peptides, inhibit bacterial proliferation by disrupting membranes and induce NETosis (153); platelets aggregate around pathogens such as S. aureus, release β-defensins, and trigger NETosis to effectively trap and neutralize the bacteria (66, 153). Similarly, thrombocidins—truncated variants of neutrophil-activating peptide-2 (NAP-2) originally isolated from platelet granules—demonstrate potent bactericidal effects against diverse bacterial strains, including *B. subtilis*, *L. lactis*, and possess fungicidal activity against *C. neoformans* (152). Moreover, platelet-secreted cytokines such as IL-1β, released upon bacterial LPS stimulation or viral infection, augment bacterial phagocytosis (3, 93, 251) and further amplify macrophage-derived IL-1β production, reinforcing antimicrobial defenses (252). Additionally, platelet-expressed GPIb modulates the fate of pathogens opsonized by complement factor C3b; typically, macrophages clear C3b-coated bacteria in the spleen, yet if platelets engage bacteria through GPIb, the platelet-pathogen complexes are redirected towards splenic dendritic cells, enhancing adaptive immune responses (248, 249). Collectively, these platelet-driven antimicrobial pathways significantly contribute to pathogen containment and the orchestration of both innate and adaptive immunity.

## Discussion

In this review, we have comprehensively outlined the intrinsic roles of platelets during infection and their corresponding functional outcomes. Platelets actively engage in immune defense by employing PRRs such as TLRs to detect pathogens (18, 19), initiating responses like immunothrombosis to limit pathogen spread (28, 29). Activated platelets undergo extensive degranulation, releasing antimicrobial peptides such as thrombocidins and β-defensins, as well as cytokines like IL-1β and various chemokines, thereby enhancing immune cell recruitment and effectively orchestrating innate and adaptive immune responses (31–34). These multifaceted mechanisms highlight platelets as central players not only in preserving vascular integrity but also in coordinating robust immune defenses during infectious states.

Platelets are essential intravascular sentinels capable of rapidly responding to pathogens or other abnormalities. Upon pathogen encounter, platelets initiate immunothrombosis, effectively restricting pathogen spread and promoting pathogen clearance through transport to immune-rich organs such as the liver and spleen (246, 247). Similarly, in malignant neoplasm pathophysiology, by shielding circulating tumor cells (CTCs) from shear stress, mediating immune evasion, and neoangiogenesis, platelets may facilitate metastasis (253), highlighting platelets’ broader biological functions beyond hemorrhage prevention and their intricate role in clinical scenarios like sepsis-associated thrombocytopenia (SAT).

### Clinical evidences and implications

Thrombocytopenia is a common complication in patients with sepsis (254, 255). Clinical data demonstrates that the prevalence of SAT varies among different research in intensive care units (ICUs), ranging from 10% to 83.5% (44, 46, 48, 256–262). Studies have shown that thrombocytopenia in sepsis patients correlates with increased mortality rates and extended ICU stays (263, 264). Furthermore, evidence indicates that persistent thrombocytopenia has an association with poor clinical outcomes (263). Recent research underscores the prognostic significance of both static and dynamic platelet indices in sepsis. For instance, Chen et al. reported that a lower platelet count (PC) and higher mean platelet volume (MPV) were independently associated with increased risks of intraventricular hemorrhage and mortality in preterm infants (265), and that transfusions at higher PCs may paradoxically increase adverse outcomes (265–267). Wang et al. identified distinct platelet trajectory subphenotypes in adult septic patients, showing that stable or declining trajectories during the first four ICU days were independently associated with increased 28-day mortality compared to ascending patterns, with thrombocytopenia mediating up to 37% of this risk (268). Cheng et al. confirmed that SAT, particularly when severe or persistent, correlates with higher in-hospital mortality in patients with sepsis-induced coagulopathy, namely, DIC (42). Similarly, Ye et al. emphasized that dynamic monitoring of platelet counts, rather than single time-point measurements, enhances the predictive accuracy for hospital mortality in sepsis, underscoring the importance of incorporating longitudinal platelet indices into clinical risk stratification models (28).

Platelet transfusion remains the most effective and widely adopted intervention to raise circulating platelet levels and is considered a cornerstone of SAT management (269–271). However, the optimal prophylactic transfusion threshold remains controversial (268, 272–274), and current clinical guidelines are hindered by the lack of high-quality evidence to support a standardized approach (275–278). Furthermore, emerging evidence indicates that a lower transfusion threshold (platelet ≤ 20x10^9^/L) may not confer significant clinical benefits in SAT patients (38, 271, 279, 280).

Regarding antiplatelet therapies, several clinical investigations have explored their potential role in infection-associated coagulopathy, particularly in the context of COVID-19. A meta-analysis involving 87,824 patients suggested that antiplatelet therapy might be associated with lower mortality in COVID-19 based on observational data (OR: 0.72, 95% CI: 0.61–0.85) (281). However, randomized controlled trials did not confirm a clinical benefit of adding antiplatelet therapy to standard care, regardless of baseline illness severity or concomitant anticoagulation (282–285).

Regarding anticoagulant therapies, recombinant human thrombomodulin (rhTM), an anticoagulant targeting excessive thrombin generation, has been widely used in Japan for sepsis-associated DIC (286). However, the SCARLET trial failed to demonstrate a mortality benefit of rhTM in a broader population with sepsis-associated coagulopathy, suggesting that the degree of coagulopathy in these patients may have been insufficient to benefit from this therapy (287). Complementary to pharmacologic strategies, Olas reviews the potential of natural phenolic compounds—such as resveratrol, curcumin, and quercetin—for their anti-platelet, antioxidant, and anticoagulant properties (288). Although these compounds show promise in modulating hemostasis and reducing oxidative stress in cardiovascular disease, their clinical relevance in COVID-19 remains speculative and largely unsubstantiated by *in vivo* evidence (288). About the immunoregulatory therapies for platelet, clopidogrel was recently used to successfully inhibit platelet inflammasome assembly and, thus, the release of pro-inflammatory IL-1β and IL-18 under septic conditions resulting in improved renal function (56, 176).

### Hypothesis

Given the complex and dynamic roles of platelets in infection revealed in this review, we propose that platelet function during sepsis is not static, but evolves in tandem with disease progression. Early in sepsis, immune hyperactivation predominates, whereas in later stages, patients may transition to an immunosuppressed state (3–5, 289, 290), often complicated by the development of DIC (1, 2, 61, 291, 292). Therefore, the predominant role of platelets likely shifts over time—from immune surveillance and modulation in early disease to consumptive coagulopathy during advanced stages. This temporal heterogeneity underpins the need for stage-specific strategies.

Based on current evidence regarding the prognostic utility of platelet indices (38, 42, 263, 268)—including absolute counts and dynamic trajectories—we hypothesize that SAT may encompass two distinct phases. Initially, platelet consumption may reflect their active participation in anti-infective and immunoregulatory processes. In patients with disease progression, however, a secondary phase may emerge where platelets are rapidly consumed due to microthrombus formation in the context of overt or subclinical DIC.

This leads to a conceptual therapeutic implication: prophylactic platelet transfusion should be considered early to preserve platelet numbers and functionality, potentially preventing irreversible transition to DIC. Interventions aimed at preserving or restoring platelet function may be more effective in this “pre-DIC window”. In contrast, antiplatelet and anticoagulant agents—although beneficial in certain coagulopathic settings—may exacerbate functional platelet inhibition that is difficult to reverse in critically ill patients. Moreover, in the context of DIC progression, platelet dysfunction induced by pharmacologic agents which are lack of reversal agents (293), may not be mitigated by transfusion alone, possibly heightening the risk of spontaneous hemorrhage (293–296).

### Limitations

Despite significant advances in understanding the intrinsic roles and functional outcomes during infection, several limitations remain that may impact the interpretation and generalizability of current findings.

First, a major constraint lies in the scarcity of human interventional data. Much of the mechanistic evidence regarding platelet immune functions originates from murine models or *in vitro* studies, which may not fully recapitulate the complexity of human immunopathology. Notably, species-specific differences in platelet receptor expression and signaling pathways can lead to divergent immune outcomes. For example, murine platelets express higher levels of TLR4—facilitating stronger responses to LPS—whereas human platelets exhibit reduced TLR4 expression and a less pronounced pro-inflammatory profile (297, 298). Moreover, discrepancies in intracellular signaling molecules (e.g., TLN1, CALM3, PRKCB in humans vs. RASGRP2, ITGB2, MYL9 in mice) may modulate platelet reactivity and immune crosstalk (299).

Second, variability in platelet preparation protocols, such as the use of different agonists in ex-vivo (e.g., thrombin, LPS), anticoagulants, and storage conditions, can influence platelet activation states and introduce inconsistencies across studies, whereas *in vivo* models like ARDS or *S. pneumoniae* infection demonstrate platelet-mediated tissue protection and resolution (300, 301). These technical variables may exaggerate or obscure specific immunological phenotypes.

Third, the inflammatory context and disease phase profoundly affect platelet function. In acute infection models such as CLP-induced or LPS-induced sepsis, platelets tend to exhibit pro-inflammatory behaviors, promoting NETosis, cytokine release, and immune cell recruitment (298, 302, 303). In contrast, chronic or resolving models—such as cancer-associated inflammation or post-viral recovery—often reveal platelet-mediated resolution and immune regulation, including promotion of Treg expansion or macrophage polarization (298, 302, 303). This functional dichotomy is not contradictory, but rather reflects the dynamic and context-dependent nature of platelet roles in immunity.

Finally, methodological heterogeneity across studies—including model choice, timing of sampling, and outcome assessment—further complicates data integration and cross-comparison.

Future investigations should aim for standardized protocols and longitudinal designs to better delineate the immunological spectrum of platelet function in infection.

### Perspectives

#### Basic and translational research

Future research, including basic and translational research, needs to emphasize the primary roles and diverse functional outcomes of platelets in driving disease progression across various clinical conditions, particularly in bloodstream infections frequently observed in sepsis. And combining acute and chronic inflammation models, and employing a diverse array of experimental techniques—from *in vivo* imaging to single-cell omics—will be essential to dissect the multifaceted roles of platelets. Particular attention should be given to the temporal dynamics of platelet activity, as their contribution likely varies across different stages of inflammation, including initiation, propagation, and resolution. Cross-species comparative studies may also provide valuable insights into the evolutionary conservation and diversification of platelet-mediated immune regulation, thereby informing both basic mechanistic understanding and translational applicability in humans.

#### Diagnose

Emerging evidence suggests that platelets, beyond their classical hemostatic functions, possess diagnostic utility in infectious diseases through their immunological responsiveness. They express a range of PRRs, including (76–79), which enable the detection of microbial components and DAMP. Differential activation of these receptors—for example, TLR4 in sepsis or TLR7 in viral infections—may serve as a cellular signature of pathogen type. Then, upon activation, platelets release immunomodulatory mediators such as PF4, CD40L, P-selectin, and IL-1β (31, 146, 174–176, 179), which are detectable in plasma and correlate with disease severity, offering potential as accessible biomarkers. Furthermore, specific pathogens induce distinct platelet responses—for instance, dengue virus activates the NLRP3 inflammasome via TLR4 (93, 94), while viruses such as HIV and HCV interact through CLRs (91, 92). These mechanistic differences may help discriminate between bacterial and viral infections.

Together, these features position platelet-derived signatures as promising diagnostic indicators for pathogen profiling, immune status assessment, and infection severity stratification.

#### Therapy

Given the central involvement of platelets in inflammation, host defense, immune. modulation, and coagulation, it is imperative to refine evidence-based protocols for prophylactic platelet transfusion, as well as to evaluate the therapeutic use of platelet-activating agents and anti-platelet drugs. As underscored by the 2022 NHLBI and OASH Transfusion Medicine State of Science Symposium (304), a deeper understanding of how donor and recipient characteristics influence not only hemostatic but also non-hemostatic platelet functions—such as immune regulation, inflammatory response, and vascular repair—may yield critical insights for clinical practice. These evolving clinical and experimental observations also support the hypothesis that transfused platelets can modulate immune responses, urging further elucidation of the molecular mediators involved in transfusion-related immunomodulation (TRIM).

Targeting the immunoregulatory functions of platelets—beyond their traditional hemostatic roles—represents a promising frontier in therapeutic innovation. Interventions that modulate platelet–immune cell interactions, such as inhibition of inflammasome assembly or disruption of platelet–leukocyte aggregates, hold potential to deliver immune benefits without compromising hemostasis (305). Notably, clopidogrel has recently demonstrated efficacy in suppressing platelet inflammasome activation, thereby reducing the release of IL-1β and IL-18 and improving renal outcomes in sepsis models (56, 176). These findings exemplify the translational promise of platelet-directed immunotherapies and highlight the need for further mechanistic and clinical exploration.

## Conclusion

In summary, platelets function far beyond hemostasis, actively bridging infection sensing, immune coordination, and pathogen elimination. These insights suggest novel avenues for immunomodulatory strategies in infection-related clinical scenarios. As our understanding of platelet immunobiology continues to evolve, targeting their immunoregulatory functions opens new avenues for diagnostics, prognostics, and therapeutics in infectious diseases. Future research should prioritize the temporal dynamics of platelet function and explore stage-specific interventions to optimize both immune support and vascular integrity in critically ill patients.
