# Supplementary material for: Platelets in infection: intrinsic roles and functional outcomes
Source: Front Immunol. 2025 Jul 7;16:1616783. doi: 10.3389/fimmu.2025.1616783 (PMC12277159; doi:10.3389/fimmu.2025.1616783)
Supplement: Supplementary file 1 [file Table1.docx]

**Supplementary File**

## The search strategies

**Table S1.** Overview of the search strategies.

| Database | Search Terms | Search Strategy |
| --- | --- | --- |
| PubMed | #1 "Blood Platelets"[mh] OR "Platelet Transfusion"[mh] OR "Plateletpheresis"[mh] OR "Platelet Aggregation"[mh] OR "Platelet Transfusion" [tiab] OR "Prophylactic Platelet Transfusion" [tiab] OR "Therapeutic Platelet Transfusion" [tiab] OR "Apheresis Platelet" [tiab] OR "Leukoreduced Platelets" [tiab] OR "Pathogen-reduced Platelets" [tiab] OR "Platelet Concentrate" [tiab] OR "Platelet Component" [tiab] OR "Platelet-rich Plasma" [tiab] OR "HLA-matched Platelets" [tiab]  #2 "Infection"[mh] OR "Infections"[tiab] OR "Infectious Diseases"[mh] OR "Systemic Infection"[tiab] OR "Bacterial Infections"[mh] OR "Viral Infections"[mh] OR "Fungal Infections"[mh] OR "Mycoses"[mh] OR "Sepsis"[mh] OR "Sepsis"[tiab] OR "Septic Shock"[mh] OR "Septic Shock"[tiab] OR "Systemic Inflammatory Response Syndrome"[mh] OR "SIRS"[tiab] OR "Endotoxemia"[tiab] OR "Bacteremia"[mh] OR "Bacteremia"[tiab] OR "Fungemia"[tiab] OR "Viremia"[tiab]  #3 "Immunity"[mh] OR "Immune System"[mh] OR "Immunologic Factors"[mh] OR "Immunomodulation"[tiab] OR "Immunoregulation"[tiab] OR "Immune Response"[mh] OR "Immune Tolerance"[mh] OR "Immune Activation"[tiab] OR "Inflammation"[mh] OR "Inflammatory Response"[tiab] OR "Cytokines"[mh] OR "Chemokines"[mh] OR "Pattern Recognition Receptors"[mh] OR "Toll-Like Receptors"[mh] OR "Toll-Like Receptors"[tiab] OR "PRR"[tiab] OR "TLR"[tiab] OR "Antigen Presentation"[mh] OR "Dendritic Cells"[mh] OR "T-Lymphocytes"[mh] OR "B-Lymphocytes"[mh] OR "Natural Killer Cells"[mh] OR "Monocytes"[mh] OR "Neutrophils"[mh] OR "Macrophages"[mh] OR "Thrombosis"[mh] OR "Thrombus"[tiab] OR "Thrombosis"[tiab] OR "Immunothrombosis"[tiab] OR "Thromboinflammation"[tiab] OR "Coagulation"[mh] OR "Coagulation"[tiab] OR "Clotting"[tiab]) OR "Transfusion-Related Immunomodulation"[tiab] OR "TRIM"[tiab] OR "Transfusion Immunomodulation"[tiab] | #1 AND #2 AND #3 |
| ISI | #1 TS=("Blood Platelets" OR "Platelet Transfusion" OR "Plateletpheresis" OR "Platelet Aggregation" OR "Prophylactic Platelet Transfusion" OR "Therapeutic Platelet Transfusion" OR "Apheresis Platelet" OR "Leukoreduced Platelets" OR "Pathogen-reduced Platelets" OR "Platelet Concentrate" OR "Platelet Component" OR "Platelet-rich Plasma" OR "HLA-matched Platelets")  #2 TS=("Infection" OR "Infections" OR "Infectious Diseases" OR "Systemic Infection" OR "Bacterial Infections" OR "Viral Infections" OR "Fungal Infections" OR "Mycoses" OR "Sepsis" OR "Septic Shock" OR "Systemic Inflammatory Response Syndrome" OR "SIRS" OR "Endotoxemia" OR "Bacteremia" OR "Fungemia" OR "Viremia")  #3 TS=("Immunity" OR "Immune System" OR "Immunologic Factors" OR "Immunomodulation" OR "Immunoregulation" OR "Immune Response" OR "Immune Tolerance" OR "Immune Activation" OR "Inflammation" OR "Inflammatory Response" OR "Cytokines" OR "Chemokines" OR "Pattern Recognition Receptors" OR "Toll-Like Receptors" OR "PRR" OR "TLR" OR "Antigen Presentation" OR "Dendritic Cells" OR "T-Lymphocytes" OR "B-Lymphocytes" OR "Natural Killer Cells" OR "Monocytes" OR "Neutrophils" OR "Macrophages" OR "Thrombosis" OR "Thrombus" OR "Immunothrombosis" OR "Thromboinflammation" OR "Coagulation" OR "Clotting" OR "Transfusion-Related Immunomodulation" OR "TRIM" OR "Transfusion Immunomodulation") | #1 AND #2 AND #3 |
| CBM | "Blood Platelets"[All fields:intelligent] OR "Platelet Transfusion"[All fields:intelligent] OR "Plateletpheresis"[All fields:intelligent] OR "Platelet Aggregation"[All fields:intelligent] OR "Prophylactic Platelet Transfusion"[All fields:intelligent] OR "Therapeutic Platelet Transfusion"[All fields:intelligent] OR "Apheresis Platelet"[All fields:intelligent] OR "Leukoreduced Platelets"[All fields:intelligent] OR "Pathogen-reduced Platelets"[All fields:intelligent] OR "Platelet Concentrate"[All fields:intelligent] OR "Platelet Component"[All fields:intelligent] OR "Platelet-rich Plasma"[All fields:intelligent] OR "HLA-matched Platelets"[All fields:intelligent] AND "Infection"[All fields:intelligent] OR "Infections"[All fields:intelligent] OR "Infectious Diseases"[All fields:intelligent] OR "Systemic Infection"[All fields:intelligent] OR "Bacterial Infections"[All fields:intelligent] OR "Viral Infections"[All fields:intelligent] OR "Fungal Infections"[All fields:intelligent] OR "Mycoses"[All fields:intelligent] OR "Sepsis"[All fields:intelligent] OR "Septic Shock"[All fields:intelligent] OR "Systemic Inflammatory Response Syndrome"[All fields:intelligent] OR "SIRS"[All fields:intelligent] OR "Endotoxemia"[All fields:intelligent] OR "Bacteremia"[All fields:intelligent] OR "Fungemia"[All fields:intelligent] OR "Viremia"[All fields:intelligent] AND "Immunity"[All fields:intelligent] OR "Immune System"[All fields:intelligent] OR "Immunologic Factors"[All fields:intelligent] OR "Immunomodulation"[All fields:intelligent] OR "Immunoregulation"[All fields:intelligent] OR "Immune Response"[All fields:intelligent] OR "Immune Tolerance"[All fields:intelligent] OR "Immune Activation"[All fields:intelligent] OR "Inflammation"[All fields:intelligent] OR "Inflammatory Response"[All fields:intelligent] OR "Cytokines"[All fields:intelligent] OR "Chemokines"[All fields:intelligent] OR "Pattern Recognition Receptors"[All fields:intelligent] OR "Toll-Like Receptors"[All fields:intelligent] OR "PRR"[All fields:intelligent] OR "TLR"[All fields:intelligent] OR "Antigen Presentation"[All fields:intelligent] OR "Dendritic Cells"[All fields:intelligent] OR "T-Lymphocytes"[All fields:intelligent] OR "B-Lymphocytes"[All fields:intelligent] OR "Natural Killer Cells"[All fields:intelligent] OR "Monocytes"[All fields:intelligent] OR "Neutrophils"[All fields:intelligent] OR "Macrophages"[All fields:intelligent] OR "Thrombosis"[All fields:intelligent] OR "Thrombus"[All fields:intelligent] OR "Immunothrombosis"[All fields:intelligent] OR "Thromboinflammation"[All fields:intelligent] OR "Coagulation"[All fields:intelligent] OR "Clotting"[All fields:intelligent] | N/A |
